# FAST MRI: DYAMOND trial protocol (can an abbreviated MRI scan detect breast cancers missed by mammography for screening clients with average mammographic density attending their first screening mammogram?)—a diagnostic yield study within the NHS population-risk breast screening programme

**DOI:** 10.1136/bmjopen-2025-106545

**Published:** 2025-09-28

**Authors:** Lyn Isobel Jones, Rebecca Geach, Abi Loose, Sadie McKeown-Keegan, Andrea Marshall, Mark Halling-Brown, Siân Curtis, Sam Harding, Jan Rose, Helen Matthews, Sarah Vinnicombe, Abeer M Shaaban, Sian Taylor-Phillips, Janet Dunn, On behalf of The FAST MRI Study Group

**Affiliations:** 1 Bristol Breast Care Centre, North Bristol NHS Trust, Bristol, Bristol, UK; 2 Bristol Medical School, University of Bristol, Bristol, England, UK; 3 Warwick Clinical Trials Unit, University of Warwick, Coventry, England, UK; 4 Scientific Computing, Royal Surrey County Hospital NHS Foundation Trust, Guildford, UK; 5 Medical Physics and Bioengineering, University Hospitals Bristol and Weston NHS Foundation Trust, Bristol, England, UK; 6 North Bristol NHS Trust, Westbury on Trym, UK; 7 Research and Development, North Bristol NHS Trust, Westbury on Trym, UK; 8 Independent Cancer Patients’ Voice, London, UK; 9 Public and Patient Representative, Bristol, UK; 10 Thirlestaine breast Centre, Gloucestershire Hospitals NHS Foundation Trust, Cheltenham, UK; 11 Histopathology, University Hospitals Birmingham NHS Foundation Trust, Birmingham, England, UK; 12 Institute of Cancer and Genomic Sciences, University of Birmingham, Birmingham, England, UK; 13 Warwick Medical School, University of Warwick, Coventry, England, UK

**Keywords:** Breast imaging, Mass Screening, Early Detection of Cancer, Magnetic resonance imaging, Breast tumours, Cancer

## Abstract

**Introduction:**

First post-contrAst SubtracTed (FAST) MRI, an abbreviated breast MRI scan, has high sensitivity for sub-centimetre aggressive breast cancer and short acquisition and interpretation times. These attributes promise effective supplemental screening. Until now, FAST MRI research has focused on women above population-risk of breast cancer (high mammographic density or personal history). DYAMOND aims to define the population within the population-risk NHS Breast Screening Programme (NHSBSP) likely to benefit from FAST MRI. The study population is the 40% of screening clients aged 50–52 who have average mammographic density (BI-RADS (Breast Imaging Reporting and Data System) B) on their first screening mammogram. DYAMOND will answer whether sufficient numbers of breast cancers, missed by mammography, can be detected by FAST MRI to justify the inclusion of this group in a future randomised controlled trial.

**Methods and analysis:**

Prospective, multicentre, diagnostic yield, single-arm study with an embedded qualitative sub-study: all recruited participants undergo a FAST MRI. An internal pilot will assess the willingness of sites and screening clients to participate in the study. Screening clients aged 50–52, with a clear first NHSBSP mammogram and BI-RADS B mammographic density (by automated measurement) will be invited to participate (recruitment target: 1000). The primary outcome is the number of additional cancers detected by FAST MRI (missed by screening mammography). A Fleming’s two-stage design will be used as this allows for early stopping after stage 1, to save participants, funding costs and time continuing to the end of the study if the question can be answered earlier.

**Ethics and dissemination:**

The NHSBSP Research and Innovation Development Advisory Committee and the Yorkshire and Humber–Sheffield Research Ethics Committee (23/YH/0268, study ID (IRAS): 330059) approved this research protocol. Participation involves a two-stage informed consent process, enabling screening for eligibility through automated mammographic density measurement. Patients with breast cancer helped shape the study design and co-produced participant-facing documents. They will disseminate the results to the public in a clear and meaningful way. Results will be published with open access in international peer-reviewed scientific journals.

**Trial registration number:**

ISRCTN74193022

**STRENGTHS AND LIMITATIONS OF THE STUDY**

- Prospective, multicentre design with defined study population and sample size determined by power calculation.
- Integrated quality assurance programme to optimise and standardise intervention image quality.
- DYAMOND First post-contrAst SubtracTed (FAST) MRI (FAST MRI) readers credentialed and certificated with an evaluated and standardised FAST MRI interpretation-training programme, using standard breast MRI interpretation benchmarks.
- Intervention acceptability to participants assessed using an embedded qualitative substudy.
- Single-arm study with limited collection of comparator data.

## Introduction

 Despite effective treatments, 30 women die from breast cancer every day in the UK.^1^ Early detection of breast cancer offers the best chance of survival^2^ and is the aim of the NHS Breast Screening Programme (NHSBSP) which offers mammographic screening to women aged 50–70 every 3 years and screens 2.12 million women/year in England.^3^

Mammographic screening programmes, including NHSBSP, result in overdiagnosis (detection of cancers which would not have caused harm during a woman’s lifetime) and underdiagnosis (failure to prevent a continued incidence of stage 2–4 breast cancers).^4 5^ Furthermore, interval cancers,^6^ which present symptomatically between screening rounds, comprise 30–44% of all breast cancers within the NHSBSP screened population.^6^,^8^ Later stage diagnosis of cancers, undetected at mammographic screening, confers a higher chance of morbidity from metastatic breast cancer and its treatment, and ultimately of mortality.^2^ This effect is compounded by mammography’s disproportionate failure to detect small, aggressive grade 3 cancers.^9^ It has been estimated that over 57 000 people in the UK are living with metastatic breast cancer and the knowledge that their cancer is incurable.^10^ Reducing breast cancer stage at detection is an important healthcare goal.

First post-contrAst SubtracTed MRI (FAST MRI) is an abbreviated breast MRI scan designed as a screening test^11^ to overcome some shortcomings of mammography, including poor sensitivity for small aggressive cancers.^911^,^13^ FAST MRI is quicker to acquire and interpret (cost and capacity benefits) than the gold standard breast screening modality, dynamic contrast-enhanced breast MRI (DCE-MRI). In the UK, DCE-MRI is currently reserved to screen only women at very high risk of breast cancer.^3 14 15^ Systematic reviews and meta-analyses have found no statistical difference in either sensitivity or specificity for cancer detection between DCE-MRI and FAST MRI, although currently a low level of evidence suggests FAST MRI could be as accurate as DCE-MRI.^13 16^ FAST MRI therefore holds promise to save more lives if used for a wider group of women than those currently screened with DCE-MRI.^11 13^

To date, research worldwide has focused on offering supplemental/additional imaging with a more sensitive imaging test (such as DCE-MRI or FAST MRI) only to women at above population risk of breast cancer, including the subset of the general population with high mammographic density.^17^,^21^ High mammographic density both increases a woman’s risk of breast cancer and increases the chances of a cancer being undetected by mammography.^17 22 23^

Worldwide, the American College of Radiologists’ Breast Imaging Reporting and Data System (BI-RADS) mammographic density classification is used to define breast composition.^24^ BI-RADS defines four density categories: A, B, C and D, where A is the least dense category and D the densest. At the age of first mammogram for population screening within NHSBSP (50–52 years), the percentage of women in each density category is approximately A: 10%, B: 40%, C: 40% and D: 10%.^25^ As breast density generally reduces in the years following the menopause,^26^ B category may be considered the ‘average’ breast density for women screened within the UK’s population-risk screening programme for breast cancer (aged 50–70).^27^

Density-determined risk-adaptive strategies that offer supplemental screening with DCE-MRI or FAST MRI, either for women with category D breasts or for those in categories C and D, result in higher cancer detection rates, a reduced incidence of stage 2 and greater breast cancers, and reduced interval cancer rates.^17 18 20 21^

However, there is insufficient evidence for the average density group (Bs) as to whether the addition of FAST MRI will be of sufficient benefit to warrant further investigation of this group in a phase III trial. This group, which makes up approximately 40% of the screened population, is excluded from other supplemental screening trials despite a knowledge gap about whether supplemental screening could benefit them.

The aim of this diagnostic yield study is to determine whether FAST MRI could detect sufficient breast cancers, missed by screening mammography, for screening clients aged 50–52 with average breast density (category B), to justify their inclusion in a future effectiveness and cost-effectiveness randomised controlled trial (RCT) of FAST MRI.

## Methods and analysis

This study protocol (Version 4.2 (26FEB2025)) is reported using the SPIRIT (Standard Protocol Items: Recommendations for Interventional Trials) reporting guidelines.^28^ Table 1 displays the study’s trial registration data.

**Table 1:** Trial registration data

Data category	Information
Trial registration	ISRCTN74193022
Date of registration	30 September 2024
Other identifying numbers	NIHR150502 (funding) NBT_5273 (sponsor), IRAS 330059 (HRA) 23/YH/0268 (REC) CPMS ID 59828 (NIHR RDN)
Source of funding	Efficacy and Mechanism Evaluation Programme, an MRC and NIHR partnership (NIHR150502)
Sponsor	North Bristol NHS Trust
Contact details	Dr Lyn Jones FASTMRI@nbt.nhs.uk
Public title	Testing whether a FAST MRI scan can detect breast cancers which were not detected on mammograms, in women with average breast density

FAST MRI, First post-contrAst SubtracTed MRI.

### Study design

FAST MRI: DYAMOND is a prospective, multicentre diagnostic yield, single-arm study of FAST MRI with an embedded qualitative substudy to examine intervention acceptability.

A 9-month internal pilot phase, from first participant recruitment, has been incorporated into the trial to assess the willingness of clinicians and screening clients to participate. The progression criteria from this phase use a traffic light system based on site opening, recruitment and compliance as detailed in Table 2.

**Table 2:** The 9-month internal pilot phase progression criteria

Threshold	Red	Amber	Green
Trial recruitment (% of the predicted recruitment at 6 months)	<65%	≥65%	≥100%
Number of sites opened	<2	≥2	4
Total number of participants recruited	<137	≥137	≥210
Compliance with FAST MRI (turning up for FAST MRI scans once booked)	<90%	≥90%	≥95%

FAST MRI, First post-contrAst SubtracTed MRI.

#### Study objectives

The primary objective is to measure the number of additional cancers detected by FAST MRI that were missed by screening mammography. The study objectives are detailed in Box 1.

Box 1 **FAST MRI: DYAMOND study objectives**

**Primary objective**

To measure the number of additional cancers detected by FAST MRI that were missed by screening mammography

**Secondary objectives**

To assess the characteristics of cancer detected by FAST MRI (including type, grade, size, stage, nodal involvement and other characteristics linked to prognosis and benefit or harm from screen detection)

To determine the acceptability of the intervention through questionnaire and an additional qualitative interview of a subset of participants

To analyse recruitment rates, retention rates, recall rates, biopsy rates, early call rates and any adverse reactions to FAST MRI

To streamline the prospective digital categorisation of mammographic density using NHS-owned software (Scientific Computing Department of the RSNFT)

To determine the proportion of screening clients in each density category (A, B, C, D) in the (UK) NHSBSP screened population at this age

To create a dataset of mammograms and corresponding FAST MRI scans with known outcome having potential use in future training and research, for example, for analysis with artificial intelligence/machine learning

To collate a central pathology database of the cancers detected for future translational research

To collect data to enable a micro-costing exercise to extend the previously conducted budget impact analysis^43^

To explore data linkage for longer term outcomes

FAST MRI, First post-contrAst SubtracTed MRI; NHSBSP, NHS Breast Screening Programme; RSNFT, Royal Surrey NHS Foundation Trust.

#### Study setting

The NHSBSP units within the UK are eligible to take part as recruiting sites, provided they can screen their clients for participation and offer a FAST MRI scan (and follow-up if required).

A list of recruiting study sites is available on the FAST MRI: DYAMOND website (https://www.nbt.nhs.uk/research-development/our-research/current-research/breast-care-research-hub/fast-mri-research-programme/taking-part-fast-mri-dyamond-study).

#### Participant eligibility criteria

Participant recruitment to DYAMOND entails a two-stage eligibility and consent process, as summarised in Box 2.

Box 2 **FAST MRI: DYAMOND two-stage consent process**

**Stage 1 Consent (breast density measurement)**

Inclusion criteria

- Age* (49 years + 8 months) ≤ (53 years – 1 day) at time of mammogram (source: NBSS)
- Has had a mammogram via the NHSBSP
- Willing and able to give informed consent to stage 1

Exclusion criteria

- Has not received a mammogram within the NHSBSP
- Aged (<49 years + 8 months) or ≥53 years at time of mammogram (source: NBSS)
- Unwilling to have mammograms measured for density
- Unwilling or unable to give informed consent to stage 1
- Breast implants

**Stage 2 Consent (FAST MRI DYAMOND scan)**

On receipt of breast density measurement for participants recruited to stage 1, participants will be screened for eligibility at stage 2.

Inclusion criteria

- First screening mammogram (prevalent round) (source: participant-reported)
- Participants with breast density category B as determined by AI breast density measurement (source: Scientific Computing Department of the RSNFT)
- Received standard NHSBSP ‘no findings’ confirmation letter (source: NBSS)
- No absolute contraindication to breast MRI (source: patient-reported, via completion of Standard Safety Questionnaire)

Exclusion criteria

- Breast density category (A, C or D or if not determined) (source: RSNFT)
- Known recall after mammogram (source: NBSS)
- Pregnant or breastfeeding
- Contraindication to MRI**
- Contraindication to gadolinium-containing contrast agents.
- Unwilling to have FAST MRI
- Unwilling to allow follow-up of outcomes through data-linkage
- Estimated glomerular filtration rate equal to or below 30 (participant reported)
- BMI, weight and abdominal girth restrictions may apply to recruiting sites’ MRI scanner(s) that could exclude otherwise eligible participants from having the study intervention

* Age range NHSBSP invites for first screening mammogram.** Participants found to have conditional contraindications to MRI will be reviewed by the local Radiology service and advice given as to the actions required to facilitate an MRI to take place, in line with local guidelines and best practice. If it is not possible to reasonably adjust for the situation in question, by following local policy, the referral to FAST MRI will be rejected and the participant will not be eligible for a FAST MRI.BMI, body mass index; FAST MRI, First post-contrAst SubtracTed MRI; NBSS, National Breast Screening System; NHSBSP, NHS Breast Screening Programme; RSNFT, Royal Surrey NHS Foundation Trust.

### Recruitment

#### Participant identification and stage 1 consent procedures

Potential participants will be identified by their usual care team at routine breast screening attendance and handed a stage 1 information leaflet at the time of their mammogram.

Advertising for the study will be present in screening clinic waiting areas in the form of posters and/or postcards. An information video will be available on the FAST MRI website, with a link/QR code to the video prominent on all advertising materials and will be shared via social media (including but not limited to Facebook and X).

Clients who wish to consent to stage 1 (consent to be screened for eligibility) can complete a consent form (paper or electronic version by following a link or QR code) and submit to the research team. Online supplemental material file 1 details both the stage 1 consent form and the stage 2 consent form.

#### Participant eligibility and stage 2 consent procedures

The research team will review the breast screening record (National Breast Screening System; NBSS) of potential participants consenting to stage 1 to check if the mammogram has been reported by the screening service and if they are aged (49 years+8 months) ≤ (53 years – 1 day). Interrogation of NBSS will be facilitated by bespoke software (created by the Scientific Computing Department of the Royal Surrey NHS Foundation Trust (RSNFT)).

Mammograms of participants who have consented to stage 1 will be measured for mammographic density, using an automated tool developed by RSNFT and results relayed to the research teams.

Participants who have been recalled by the standard mammographic screening process for further investigations but are otherwise eligible will be notified that they are not eligible to have a FAST MRI but are still eligible for data linkage. Participants who are ineligible for any other reason will be contacted and the reason for ineligibility will be explained.

Participants who are breast density B and remain potentially eligible will be identified and sent the stage 2 study information. The remaining inclusion and exclusion criteria will be confirmed with the participant during the stage 2 Informed Consent procedure.

Eligible participants consenting to stage 2 will be booked for a FAST MRI appointment.

**Figure 1:**
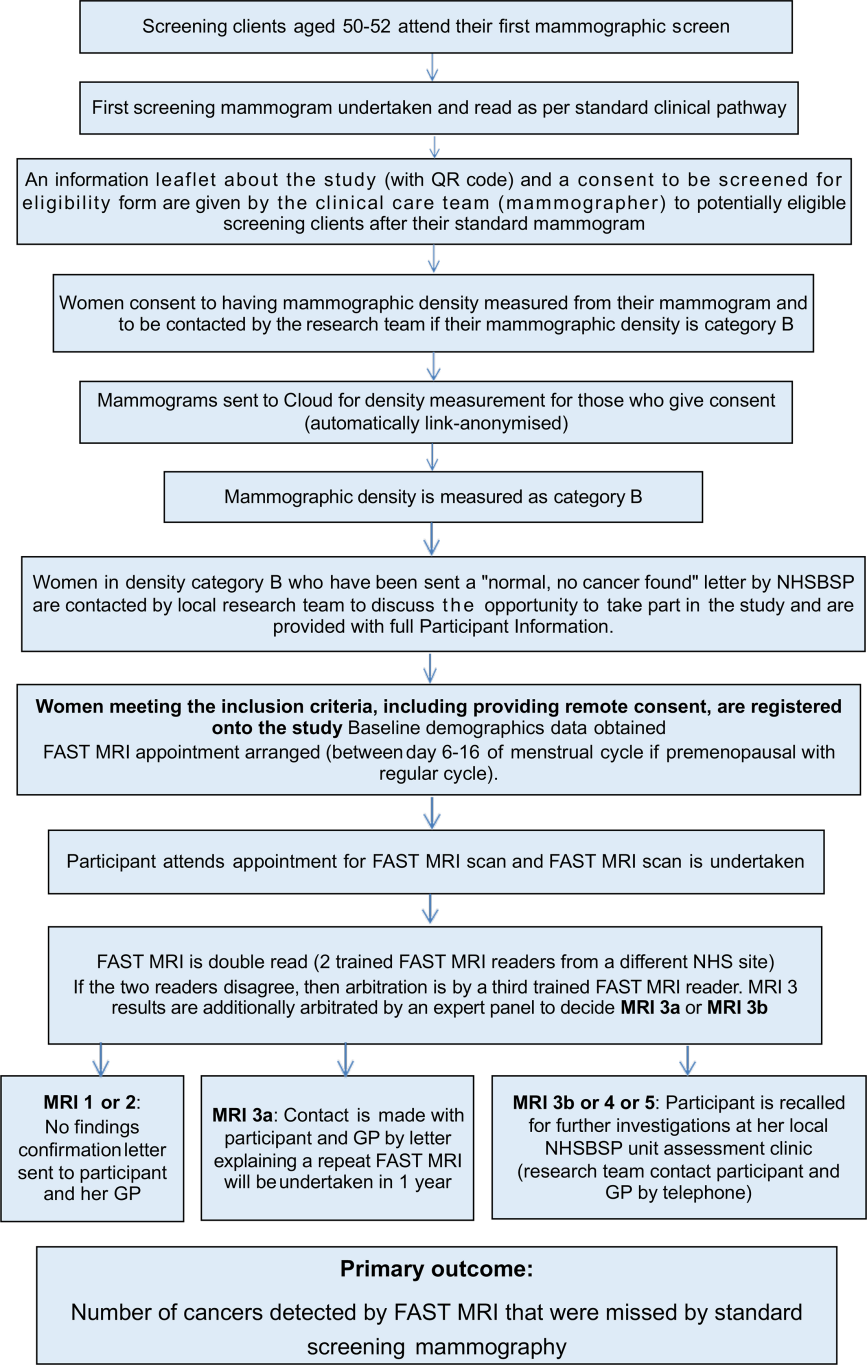
Study flow chart. FAST MRI, First post-contrAst SubtracTed MRI; NHSBSP, NHS Breast Screening Programme.

The study flow chart is reproduced in Figure 1.

#### Intervention

The study intervention is a FAST MRI scan (**F**irst post-contr**A**st **S**ubtrac**T**ed images of a dynamic contrast-enhanced breast MRI scan).^11^ All standard MRI safety protocols will be followed, including the completion of a safety checklist to ensure that there are no MRI contraindications.

#### Scanning protocol parameters

The scanning protocol parameters will be within those set out for the DCE-MRI sequences of standard breast MRI by the NHSBSP as technical guidance for screening women at very high risk of breast cancer with MRI.^14^ The exact parameters used will be determined for each make and model of scanner (3T or 1.5T) by DYAMOND’s Central Quality Assurance Team during site set-up.

#### Quality assurance (QA) of FAST MRI scans

Dedicated FAST MRI QA phantoms (test objects)^29^ and an MRI equipment QA protocol have been developed for the DYAMOND study (to be published as a separate paper) for the purpose of optimising and standardising the FAST MRI scanning protocols and performing QA of the clinical FAST MRI scans at all study sites. All sites and scanners must pass the QA phase prior to greenlight for recruitment to the main study. This process enables quality checking of images and data centrally by the study QA Team.

#### FAST MRI interpretation and display

FAST MRI scans acquired during the study are displayed as maximum intensity projections and stacks of slices in axial and sagittal planes.

FAST MRI readers are both researchers and study participants (FAST MRI reader substudy).

DYAMOND FAST MRI readers must:

- Be employed by a participating site.
- Be a mammogram reader for the NHS Breast Screening Programme.
- Have completed FAST MRI DYAMOND reader training.^30^,^34^
- Have credentialed (by interpreting a standardised assessment test set of FAST MRI scans with known outcome and achieving a predefined specificity and sensitivity).^34 35^
- Have completed Good Clinical Practice (GCP) training and be included on the site delegation log.
- Be able and willing to provide their informed consent.

FAST MRI readers are trained to classify scans according to the UK 5-point scale^14 36^ (MRI 1: normal; MRI 2: benign; MRI 3: indeterminate/probably benign; MRI 4: suspicious of malignancy; and MRI 5: highly suspicious of malignancy).

The FAST MRI scans will be interpreted by at least two certified FAST MRI DYAMOND readers, with arbitration for disagreement between readers.

The readers will be from a site other than that of the participant whose scan is being interpreted to ensure that they have no access to the participant’s standard care mammogram when interpreting the FAST MRI. This will be achieved using image viewing technology developed by RSNFT (RiViewer).^37^

All MRI 3 results will be reviewed by an expert panel who will additionally classify the scan as 3a or 3b.

#### FAST MRI results

If a participant’s FAST MRI scan is classified as:

- MRI 1 or 2,^36^ the participant and her General Practitioner (GP) will be sent a normal findings confirmation letter.
- MRI 3a, the participant and GP will be sent a letter telling them that they will be invited for a repeat FAST MRI in 1 year (Y1 FAST MRI scan).
- MRI 3b, 4 or 5,^36^ the research team will contact the participant by telephone to inform them of an appointment for further investigation.
  - Those found to have a biopsy-confirmed breast cancer (invasive and non-invasive) will be treated as per local practice appropriate for the size, stage and type of cancer detected.
  - Those with no cancer confirmed will be invited for a repeat FAST MRI in 1 year (Y1 FAST MRI scan).

At year 1, scans will be classified into MRI 1/2 (no further action required) or MRI 4/5 with a referral to the assessment clinic. Readers at year 1 will have access to the year 0 scans for reference/comparison.

### Study outcomes, statistical analysis, data collection and data storage

#### Study outcomes

The primary outcome is the number of cancers (invasive and non-invasive) that are detected on FAST MRI, for participants whose FAST MRI scans were classified as MRI 3b, 4 or 5, after a negative mammogram.

Secondary outcomes are as follows:

- The characteristics of the cancers detected by FAST MRI (including type, grade, size, stage, nodal involvement).
- Acceptability of the intervention through questionnaire and an additional qualitative interview of a subset of participants.
- Recruitment rate: Those that are recruited out of all those with density B and a clear mammogram that were contacted.
- Retention/compliance rate accounts for the number that withdraw from the study once recruited or do not attend the FAST MRI scan.
- Recall rate: The proportion of participants classified with a MRI 3b, 4 or 5 and invited for further investigation out of the total number of recruited participants who had a FAST MRI.
- Biopsy rate: The number of biopsies undertaken out of all participants recruited who had a FAST MRI.
- Early call rate: The number of participants who have a FAST MRI at Y1 (includes all those classified as MRI 3a, and those classified as MRI 3b, 4 and 5 who did not have cancer confirmed at Y0 biopsy) out of all participants recruited who had a FAST MRI at Y0.
- Adverse reactions to FAST MRI.
- Proportion of screening clients in each density category (A, B, C, D) of those screening clients consenting at stage 1.
- Interval cancers detected during the study period.
- FAST MRI classifications of Y1 scans (for all those with MRI 3a at Y0 and those with MRI 3b, 4 and 5 and no cancer detected at Y0).
- FAST MRI reader data, diagnostic accuracy of readers within the study (individually and by reader group and NHS site), numbers of scans requiring arbitration, standard arbitration outcomes and expert panel arbitration outcomes.

Cancer registry and NBSS records will be reviewed during the study to identify screen-detected or interval cancer data for all those taking part in the study including the data link participants (screening clients who consented at stage 1 with BI-RADS B mammographic density but did not have a FAST MRI scan for any reason and have not opted out of data linkage). This review will help to identify cancers that were screen detected at standard care mammography and any interval cancers that occur during the study for the screening clients who consented at stage 1, including those who also consented at stage 2.

#### Analysis of feasibility and uptake

Analysis of activity timings and comparison with the trial assessment schedule will provide insight into capacity with regard to MRI scanning, breast radiology workforce and screening assessment clinic provision within the NHS.

#### Micro-costing exercise

Data will be collected for a micro-costing exercise to enable estimates of cost for the intervention and for assessment clinics, biopsies and breast cancer treatments.

### Statistical analysis

#### Sample size calculation

From previous studies, in population-risk screening clients aged 50–54 of all mammographic densities having their first screening mammogram (prevalent round), standard mammography finds approximately 7.5 cancers per 1000 women screened^38^ (there are no reported figures specific to age 50–52). The addition of MRI may detect an additional 16.5 per 1000 women screened^20^ in women with mammographically dense breasts (density category D^24^). Meta-analyses have shown that sensitivities and specificities for FAST MRI did not differ significantly from those for DCE-MRI,^13 16^ but confidence in the evidence was low and FAST MRI may not pick up as many cancers as DCE-MRI.^9^

A Fleming’s two-stage design^39^ will be used to assess the number of additional cancers detected by FAST MRI. This design allows for early stopping after stage one, which would save patients, funding costs and time continuing to the end of the study if the question could be answered earlier. Based on previous research and consensus from radiologists, health economists and public and patient involvement (PPI) representatives, 4 additional cancers detected per 1000 women screened was deemed the minimum that would be required to be detected to warrant further investigation of this study population in a phase III trial. Setting the lower limit for the cancer detection at 0.004 (4 additional cancers detected per 1000 women screened), the target rate at 0.011, 80% power and a 5% one-sided significance level, then a minimum of 959 participants would be required (381 participants at the first stage). Thus, recruiting 1000 participants to the study will allow for 4% dropouts.

An interim analysis will be performed when 381 participants have been recruited. If one or no additional cancers are detected, the study may be halted for futility. If five or more cancers are detected, then the study may be stopped early as it has already been shown that the inclusion of this population in a phase III trial is warranted. Otherwise, the study will continue, and eight or more additional cancers are required to be detected by the end of the study to warrant inclusion of this population in a future phase III trial.

#### Data analysis plan

The primary outcome of the number of cancers detected on FAST MRI after a negative mammogram will be converted into a rate per thousand women screened to be comparable with current evidence and the associated 95% CI determined.

Summary statistics of the participant characteristics, tumour characteristics and adverse events will be presented as counts and percentages for categorical variables and means and SD or medians and IQRs for continuous variables depending on the distribution of the data.

The recruitment, compliance, recall, early call, biopsy and interval cancer rates, together with associated 95% CIs, will be determined.

This is a single-arm study, and therefore, no formal statistical testing is expected. All participants registered into the study will be analysed where possible. Cancer detection rates and adverse events will be analysed using the as treated population that had a FAST MRI. Participants who subsequently withdrew consent to participate will be analysed up until the point of withdrawal. To limit missing data, participants’ involvement is kept to a minimum with individuals only expected to attend for a FAST MRI and subsequent investigation depending on the result of the FAST MRI. Data collection is through the screening services and hospital data.

### Data collection and storage

#### Main database at Warwick Clinical Trials Unit

Electronic case report forms for potential participants’ pseudonymised data once stage 1 consent is completed, to be held on a dedicated database hosted by Warwick Clinical Trials Unit on a secure server with restricted access. This data will include demographic data and also clinical and particularly breast history, risk factors for developing breast cancer (including Tyrer-Cuzick score) and menopause status.

#### Image database at Royal Surrey NHS Foundation Trust Scientific Computing Department (RSNFT)

Stage 1 participants’ pseudonymised mammograms (stage 1 consent completed)±FAST MRI scans (stage 2 consent completed) will be held on a dedicated image database hosted by the RSNFT.

Images are transferred via the Image Exchange Portal (IEP) to a FASTMRI node (hosted at RSNFT). The images are deidentified as they transition from IEP, meaning that no patient identifiable information is received outside the IEP network. On upload, a pseudonym will be assigned to the patient. This will be created by a lossful encoding algorithm and complex salt which will produce an encoded pseudonym that allows the linking of the screening client’s trial ID with the images.

**Figure 2:**
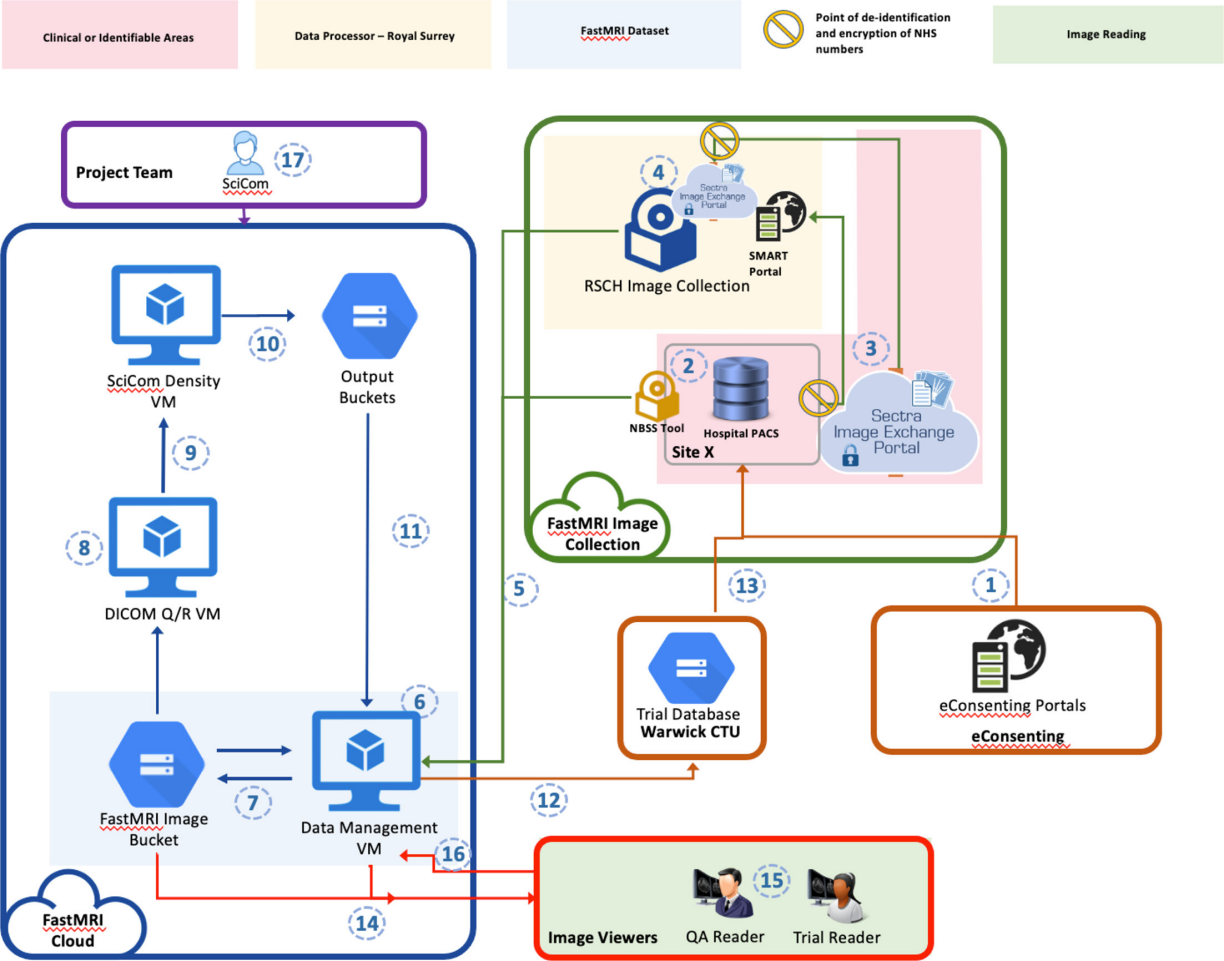
Data flow. FAST MRI, First post-contrAst SubtracTed MRI. (1) Clients use the eConsenting portals to consent to stage 1 and stage 2 of the trial. (2) Local; sites use the NBSS tool to produce a screening log. (3) Client’s images are acquired, and the local site registers the clients on the SMART web portal before transferring the images via IEP to the RSCH dedicated research node. (4) Images are received at the RSCH IEP node and processed through a pipeline to pseudonymise and prepare, then. (5) Images are pipelined and routed to the relevant staging areas. (6) Images arrive in a data management staging area in the dedicate cloud project. (7) Once images are processed by the data management staging area, they are routed to a temporary storage area (buckets). (8) A DICOM QR node exists in the dedicate cloud whose role is to monitor the incoming images into the temporary storage, and (9) Route the images to the Breast AI tool (10) Results from the Breast AI tool are routed to an output bucket (11) Results from the Breast AI tool are staged to the Data Management area (12) Results from the SciCom activities are routed to the Warwick database via dedicated APIs (13) The trial database is accessed by central DYAMOND research team and recruiting sites (14) Images are collected into reading sessions and staged in the RiViewer staging area. (15) Reading sessions are completed by various trial readers. (16) Results from reads are routed to the Data Management area and thence to the trial database. (17) SciCom team monitors these processes.

The FAST MRI: DYAMOND data flow diagram is presented in Figure 2.

### Substudies

#### Substudy to assess the acceptability of the intervention

The aim is to gain greater understanding of the impact of the research process and the acceptability of FAST MRI.

Each participant who has consented to stage 2 will be asked for feedback on acceptability relating to the FAST MRI scan via a survey. The survey is detailed in online supplemental material file 2.

Qualitative data collected on the survey will be analysed using qualitative content analysis. The survey will give the participant the option to agree to be contacted to take part in an interview to discuss their answers further. Participants who agree to be contacted for interview will be sent a qualitative study information sheet. Informed consent will be received verbally and audio-recorded at the start of the arranged interview, and the researcher will complete the informed consent form in writing on the participant’s behalf.

Demographic information, including about protected characteristics, will be collected for all participants at the time of the stage 2 consent (to FAST MRI) process. This information, along with results of the analysis of the survey, will be used to purposively select potential participants for the qualitative interviews, to assess the equality and equity impact of the research. If inequalities are identified while this study is ongoing, amendments to process will be investigated and discussed with the steering committee and the PPI panel under the guidance of the study’s PPI leadership team. If deemed feasible, changes may be rolled out following obtaining appropriate approvals.

Semistructured interviews with up to 60 participants will be undertaken. Interviews will be subject to a priori framework analysis using the Theoretical Domains Framework (TDF).^40 41^ The topic guide for the interviews is detailed in online supplemental material file 3.

#### FAST MRI reader substudy

We will report FAST MRI reader outcome data (by professional group, experience and demographics data), diagnostic accuracy of reader groups, time taken to report scans, numbers of scans requiring arbitration, standard arbitration outcomes and expert panel arbitration outcomes.

An additional analysis, for scans classified as MRI 3, will compare expert panel decisions (3a or 3b) versus standard reader opinion (3a or 3b) with scan outcome at study end (cancer/no cancer).

#### Central pathology collation of cancers detected

At the time of stage 2 consent, the research nurse will explain to all participants that if they should be recalled for further investigation following their FAST MRI scan, there will be an option for additional tissue analysis and donation. This information is also included in the stage 2 Participant Information Sheet.

A separate Tissue Donation Participant Information Sheet and Consent Form will be offered to those participants recalled for further investigations from their FAST MRI scan. Then, at the assessment clinic, participants will be approached by their clinical team or a member of the research team to ask for consent for a tissue sample to be sent to the University of Birmingham (UoB) for additional analysis and potential donation to the UoB tissue bank.

Participants who consent will donate an additional sample at the time of their percutaneous biopsy. If subsequent routine histological analysis of the biopsy shows it to be a cancer, the additional research sample will be examined to ensure the sample contains cancer tissue and then undergo additional analysis and be stored for future research.

Central review of cancer cases by an expert specialist histopathologist will comprise:

- Histological review to confirm type and grade of cancer.
- Molecular categorisation, for example, luminal, triple negative, HER2 positive cancer.
- Scoring of tumour-infiltrating lymphocytes (TILs) following the guidelines of the International Immuno-Oncology Working Group.^42^

Should additional funding become available during the study, further analysis will be undertaken to profile the microenvironment.

After all study analysis has been undertaken, if the participant has consented, then the samples will be gifted to the Human Biomaterials Resource Centre at the University of Birmingham for future research.

### Public and patient involvement (PPI)

Our PPI contributors include external patient groups, PPI leadership team and PPI panel as detailed in Box 3 and are a vital and respected part of the FAST MRI Research Team. Our collective PPI contributors’ personal experiences enabled them to guide and question the research team during the planning of the research question and study design.

Box 3 **Public and patient involvement (PPI)**

**PPI contributors to this study**

PPI leadership team: Two PPI lay researchers and one PPI lead/facilitator

External patient groups:

ICPV: https://www.independentcancerpatientsvoice.org.uk/

BUST: https://www.bustbristol.co.uk/

UK Breast Cancer Clinical Studies Group: https://breastcancernow.org/sites/default/files/cp_uk_bccsg_position_paper_tor_-_final_mar_2024_1.pdf

Breast Density Matters UK: https://www.facebook.com/breastdensitymattersuk/

Lobular Breast ancer UK: https://lobularbreastcancer.org.uk/

PPI Panel: A dedicated panel (of 6 plus 2 Lay Researchers from the PPI Leadership Team) identified from: individuals having breast screening with mammography and/or MRI and women with lived experience of breast cancer

**PPI contribution to study design**

Our PPI contributors guided the design of:

Recruitment strategies:

- Concept of an infographic/visual flyer and poster
- First approach by the clinical team at standard mammogram attendance
- Development of a two-stage consent process

Methods of results delivery:

- Positive results to be delivered by telephone—by someone able to answer questions about what the result will mean for the participant
- Negative results to be delivered by letter/email

Methods to reduce false-positive biopsies:

- Importance of explaining the results categories to participants at the outset (verbal and written information)

**PPI contribution to study conduct**

At study set-up, PPI helped to ensure the study protocol is feasible, inclusive and accessible to study participants. Additionally, the design, accessibility and inclusivity of the participant information, including the formal participant information sheet, was written jointly with the two lay researchers and reviewed by the wider PPI Team.

During the study, the PPI team will work with the study’s qualitative researcher to support the analysis and collaborate on the interpretation of the qualitative results to help validate the results and to inform future study design and any changes required for the current study.

**PPI contribution to study dissemination**

Evaluation of our results by the public through the external patient organisations within our PPI network will be vital since important questions about the acceptability of levels of cancer detection and of error in screening are likely to be raised.

Our PPI contributors will lead public dissemination of the study’s progress and results, ensuring communications, publications and presentations are accessible and engaging.

Our PPI contributors were central to the design of the participant-facing elements of the proposed research. They have highlighted the importance of keeping MRI scan times short and that, for them, the absence of ionising radiation is an important advantage of FAST MRI over mammography. They expressed their appreciation of the fine balance of risk versus benefit in breast cancer screening.

The clinicians and PPI team discussed the literature and estimates of false-positive biopsies from FAST MRI and collaboratively developed the study’s strategy for dealing with MRI 3 (uncertain/probably benign) results, taking into account how these might be interpreted when reported to participants.

Our PPI leadership team have co-produced all participant-facing literature and advertisements.

The PPI group will be involved in the interpretation of the interview data (intervention acceptability substudy) to check research interpretation. PPI members are part of the study management group and trial steering committee. They will be involved in the study conduct and are integral to plans for dissemination of the results (Box 3).

## Ethics and dissemination

### Ethical considerations

The study will be conducted according to the UK Policy Framework for Health and Social Care research and to the international standards known as ‘Good Clinical Practice’.

The NHSBSP Research and Innovation Development Advisory Committee approved the protocol on 27 September 2023.

This study was approved by the NHS Health Research Authority (HRA) and Health and Care Research Wales (HCRW) following a favourable ethical opinion being received from Yorkshire and The Humber–Sheffield Research Ethics Committee (REC) on 9 January 2024 (REC reference 23/YH/0268 IRAS 330059).

### Trial oversight

The Trial Management Group (TMG) is responsible for the daily running and management of the study.

The Independent Data Monitoring Committee (IDMEC) is responsible for reviewing and assessing recruitment, interim monitoring of safety and effectiveness, trial conduct and external data, via the IDMEC Report to the Trial Steering Committee (TSC).

The TSC provides advice to the TMG, funder and the Sponsor via the Independent Chair and is responsible for the ultimate decision for the continuation of the study.

### Safety considerations

This trial is categorised as: Type B=Somewhat higher than the risk of standard medical care. The study intervention (FAST MRI) includes the use of gadolinium-based contrast agent (GBCA), which poses a medium level of risk. The risk of GBCA cannot be mitigated; however, safety protocol in relation to adverse events is well documented. The risk and potential adverse impacts from having a FAST MRI scan are detailed in online supplemental material file 4.

### Key study dates

Greenlight for recruitment start: 4 April 2025

Recruitment of first participant (time of first FAST MRI scan): 22 May 2025

Recruitment is anticipated to take 8 months to the recruitment target of 1000 participants.

Grant end date is 31 October 2026.

### Dissemination

Planned publications:

- Study protocol (this report).
- FAST MRI acceptability.
- Primary outcome.

Results will be published using open access formats in international peer-reviewed scientific journals.

#### Data storage, curation and archiving

Approval was granted by the HRA and REC for the data storage, curation and archiving methods stipulated in the study protocol.

## Discussion

Defining the population likely to benefit from an intervention is an essential step in the design of a full effectiveness and cost-effectiveness trial. This study will discover whether screening clients with average breast density could potentially benefit from breast screening with FAST MRI and the results of this study will inform the design and study population of a future RCT to assess FAST MRI’s effectiveness and cost effectiveness as a screening tool for breast cancer within the NHSBSP’s population-risk mammographic screening programme.

The current study will explore QA processes utilising novel, dedicated phantoms and an equipment QA protocol to standardise FAST MRI quality across NHS sites. It will answer uncertainties around feasibility, acceptability, uptake, recall rates and the differential use of imaging modalities needed to guide biopsies indicated by FAST MRI (ultrasound, tomosynthesis and MRI). Evaluation of this study’s novel method of image analysis for uncertain/probably benign results (MRI 3 classifications) will inform the method of image analysis that will be used in the future RCT.

The aim of the FAST MRI research programme (https://www.nbt.nhs.uk/FASTMRI), sponsored by North Bristol NHS Trust, is to investigate whether FAST MRI can cost-effectively detect aggressive cancers earlier than current standard (screening) practice and thereby reduce both breast cancer mortality and the number of people experiencing years of morbidity from living with metastatic breast cancer. The programme has great potential for positive impact. This current study is an important step to define who could benefit from screening with FAST MRI.

## Supplementary material

10.1136/bmjopen-2025-106545 online supplemental file 1
