# Supplementary material for: FAST MRI: DYAMOND trial protocol (can an abbreviated MRI scan detect breast cancers missed by mammography for screening clients with average mammographic density attending their first screening mammogram?)—a diagnostic yield study within the NHS population-risk breast screening programme
Source: BMJ Open. 2025 Sep 28;15(9):e106545. doi: 10.1136/bmjopen-2025-106545 (PMC12481369; doi:10.1136/bmjopen-2025-106545)
Supplement: online supplemental file 1 [file bmjopen-15-9-s001.pdf]

Supplementary material file 1: Consent forms at Stage 1 and Stage 2

FASTMRI DYAMOND

NHS North Bristol NHS Trust

SCREENING CLIENT: CONSENT TO BE CONTACTED TO ASSESS ELIGIBILITY

FAST MRI: Diagnostic **Y**ield study for **A**verage **M**amm**O**graphic scree**N**ing **D**ensity

The FAST MRI DYAMOND Study – Stage 1

Thank you for reading the DYAMOND Stage 1 information leaflet and for considering participation in this important research. If you would like to take part in Stage 1 of the FAST MRI DYAMOND Study (to see if you can have a FAST MRI in the future) please complete the following consent form.

If you have **not** received an Information Leaflet about the FAST MRI DYAMOND Study (Stage 1), or if you would like to discuss it with a researcher, please contact your local research team on XXXX

Your full name (please print)										
Your date of birth	_____/_____/______									
Date of your mammogram	_____/_____/______									
Time of your mammogram appointment										
Place of your mammogram appointment										
Your NHS Number (if known)										

If Stage 1 shows that you are eligible to take part in Stage 2, please let us know how you would like to be contacted:

Email	Yes / No	Please give preferred email address:	
Post	Yes / No	Please give preferred postal address:	
Text	Yes / No	Please give preferred mobile telephone number:	
Phone	Yes / No	Please give preferred telephone number (if different from above):	
		Preferred days to be phoned:	Preferred times to be phoned:

Please turn over and complete Page 2

Please <u>initial</u>

all boxes

1	I confirm that I have read the Information Leaflet about the FAST MRI DYAMOND Study (Stage 1), version X (dated XX/XX/XX). I have had the opportunity to consider the information and to ask questions and I have had these questions answered satisfactorily	
2	I consent for members of the FAST MRI DYAMOND study team to send my mammogram to NHS researchers at the Royal Surrey NHS Foundation Trust for them to use a computer to grade my breast density	
3	I understand that my mammogram will not be sent until 10 working days after my consent has been received and that I can contact the study team within this time if I no longer wish for my breast density to be graded	
4	I understand that my mammogram and my data will be shared and stored electronically with researchers at Warwick Clinical Trials Unit and Royal Surrey NHS Foundation Trust	
5	I consent for members of the study team to look up the results of my recent mammogram and to access my screening data and breast care records electronically even if I am unable to have a FAST MRI scan	
6	I understand that I may withdraw my consent at any time and my clinical care will not be affected	
7	I understand that if the results of Stage 1 show that I am **not eligible** to take part in Stage 2 of the study I will be contacted by the Research team by letter and that my care will continue with the Breast Screening Service and / or local Breast Care service	
8	I understand that relevant sections of my medical notes and data collected during the study may be looked at by individuals from North Bristol NHS Trust (who Sponsor the study), by regulatory authorities or by other members of the research team, where it is relevant to my taking part in this research. I give permission for these individuals to have access to my records	
9	I understand that if the results of Stage 1 show that I am **eligible** to take part in Stage 2 of the study, I will be contacted by the research team with some information about having a FAST MRI DYAMOND scan.<br><br>I consent to take part in Stage 1 and to be contacted by a member of the research team.	

Name of Screening Client (please print)	Signature of Screening Client	Today's Date

Thank you for completing this form. Please return this form to the research team (localise to method of

Please turn over and complete Page 3

To be completed by the Researcher:

Participant ID Number		Participant Initials	

Countersigned by Researcher (please print name)	Signature of Researcher	Date

1 copy to participant | 1 copy to Site File | 1 copy to database

FAST MRI: Diagnostic **Y**ield study for **A**verage **M**amm**O**graphic scree**N**ing **D**ensity

The FAST MRI DYAMOND Study – Stage 2 Consent Form

Thank you for reading the DYAMOND Stage 2 information leaflet and for considering participation in this important research. If you would like to take part in Stage 2 of the FAST MRI DYAMOND Study, please complete the following consent form.

If you have **not** received an Information Leaflet about the FAST MRI DYAMOND Study (Stage 2), or if you would like to discuss it with a researcher, please contact your local research team on xxxxx

Please initial all boxes

1	I confirm that I have read the information sheet dated.................... (version...........) for the above study. I have had the opportunity to consider the information, ask questions and have had these answered satisfactorily	
2	I understand that my participation is voluntary and that I am free to withdraw consent at any time without giving any reason, without my medical care or legal rights being affected	
3	I understand that relevant sections of my medical notes and data collected during the study, may be looked at by individuals from North Bristol NHS Trust (who sponsor the study), regulatory authorities, or the NHS Trust, where it is relevant to my taking part in this research. I give permission for these individuals to have access to my records	
4	I give permission for data collected about me, including images (MRI and mammogram) to be used to support further training for others and ethically approved research in the future. I understand that nobody will be able to identify me from these data	
5	I agree to my General Practitioner (GP) being informed of the results of my FAST MRI scan	
6	I understand that I may be invited for further clinical investigations which may include x-rays and understand the risks associated with this. These have been outlined in the Participant Information Sheet	
7	I understand that information held by the NHS, including by the Breast Screening Programme and the Cancer Registry may be used to provide information about my health status and I give permission for long term anonymised storage and use of this and other information about me, for health-related research purposes only (even after my incapacity or death	
8	I agree to take part in the above study	

Participant's Name (please print)	Participant's Signature	Today's Date

To be completed by the Researcher

Participant ID Number		Participant Initials	
Study Site			

Countersigned by Researcher (please print name)	Signature of Researcher	Date

1 copy to participant | 1 copy to Site File | 1 copy to database

Supplementary material file 2: Acceptability survey

**NHS number**										
**Participant ID**										

FASTMRI DYAMOND

Questionnaire: How was it for you?

Thank you for taking part in the FAST MRI DYAMOND Study and for having a FAST MRI scan.

As mentioned in your Participant Information Sheet, it is very important to us to get your feedback on the FAST MRI scan you have just had.

Please complete this short survey as soon as you can after your scan. The questionnaire should take no longer than 10 minutes to complete but if you have any questions, please do not hesitate to ask a member of the team using the contact details on the back of the study information sheet.

If you would like to complete this online, please scan this QR code, or visit https://app.onlinesurveys.jisc.ac.uk/s/nbt/dyamond-qualitative-survey

Alternatively, you can complete on paper and hand to a member of the team today [may be removed when localised if facilities to collect paper surveys do not exist]

Date of completion: __ __ / __ __ __ / __ __ __ __

About you

Q1. Have you had any sort of MRI scan before today? (please tick all boxes that apply)

This is my first MRI ☐ Breast ☐ Other MRI ☐

Your experience of the scan

Q2. After your mammogram, how long did you have to wait until you heard your results?

__ __ days

NHS number										
**Participant ID**										

Q3. How long after your mammogram did you receive your invitation for your FAST MRI scan?

__ __ days

Q4. Was there anything you particularly LIKED about the invitation and information you were sent about the FAST MRI scan? (please write below)

Q5. Was there anything you particularly DISLIKED about the invitation and information you were sent about the FAST MRI scan? (please write below)

Q6. How could the information you received about the scan be improved? (please write below)

Q7. Were you given enough choice about when you could schedule your FAST MRI scan?

Yes ☐ No ☐

If No, how could we improve the scheduling options for the FAST MRI scan (please write below)

Q8. During your visit to have the FAST MRI scan, how often did you feel you had a chance to discuss any concerns you had?.

Never ☐ Sometimes ☐ When necessary ☐ Frequently ☐ Always ☐

NHS number										
**Participant ID**										

Q9. During your visit to have the FAST MRI scan, did you feel treated with dignity and respect?

Never ☐ Sometimes ☐ When necessary ☐ Frequently ☐ Always ☐

Q10. During your visit to have the FAST MRI scan, did you think that the clinical team knew all the important information about you and the scan you were scheduled to have?

Yes ☐ No ☐

If No, what information do you think they needed, but did not have? And/or what information did you expect them to have that they did not have? (please write below)

Q11. During your visit to have the FAST MRI scan, did anything happen that you weren't expecting?

Yes ☐ No ☐

If Yes, could you please tell us what? (please write below)

Q12. During your visit to have the FAST MRI scan did you feel that you were being given a high quality and safe service?

Never ☐ Sometimes ☐ When necessary ☐ Frequently ☐ Always ☐

Q13. During your visit to have the FAST MRI scan, did you feel like you had an understanding of the benefits of the scan?

Never ☐ Sometimes ☐ When necessary ☐ Frequently ☐ Always ☐

Q14. During your visit to have the FAST MRI scan, how long did you have to wait before having your scan?

__ __ minutes

NHS number										
**Participant ID**										

Q15. How did the wait feel?

The wait was too long ☐

The wait was acceptable ☐

The wait was too short ☐

Q16. Please tell us anything that could be improved about the service environment e.g., quality of transport links, availability of park, cleanliness of the clinic, level of noise or distraction in the waiting area, before during or after the scan. (please write below)

Acceptability

Q17. Compared to what you were expecting before your scan, how was your experience of a FAST MRI today?

Much worse than expected	A little worse	About the same	A little better	Much better than expected
☐	☐	☐	☐	☐

Q18. After only mammograms, do you trust that the findings (that you do or do not have breast cancer) are correct?

No trust	Low level of trust	Barely acceptable	Good level of trust	Very good level of trust
☐	☐	☐	☐	☐

**NHS number**										
**Participant ID**										

Q19. After only FAST MRI, do you trust that the findings (that you do or do not have breast cancer) are correct?

No trust	Low level of trust	Barely acceptable	Good level of trust	Very good level of trust
☐	☐	☐	☐	☐

Q20. Please rate your level of concern that the mammogram might miss an important breast cancer

No concern	Little	Moderate	Intense	Very intense
☐	☐	☐	☐	☐

If you are concerned, why are you concerned? (please write below)

Q21. Please rate your level of concern that the FAST MRI scan might miss an important breast cancer

No concern	Little	Moderate	Intense	Very intense
☐	☐	☐	☐	☐

If you are concerned, why are you concerned? (please write below)

Q22. I prefer screening:

- ☐ With mammogram
- ☐ With FAST MRI
- ☐ With both mammogram and FAST MRI
- ☐ No preference
- ☐ No screening at all
- ☐ Other (if other, please write what you would prefer below)

NHS number										
**Participant ID**										

Are there any additional comments you would like to make? (please write below)

Contacting you

Would you be willing to tell us more about your experiences of having a FAST MRI?

☐ Yes
☐ No

Please provide an email that we can use to contact you (please print)

____@____

Please provide a telephone number that we can use to contact you

____

Please indicate the usual best times and days to contact you.

Monday	☐	AM	☐
		PM	☐
Tuesday	☐	AM	☐
		PM	☐
Wednesday	☐	AM	☐
		PM	☐
Thursday	☐	AM	☐
		PM	☐
Friday	☐	AM	☐
		PM	☐

If there is a specific day and time, please write below. We can't guarantee we'll be able to contact you at these times, but will take it into consideration before contacting you.

Thank you for completing this survey, and for participating in the DYAMOND study.

Supplementary material file 3: Interview topic guide

FASTMRI DYAMOND

NHS
North Bristol
NHS Trust

Interview schedule

[Overview of the project, and verbally check consent]

Experience of taking part in research

Tell me about how you found out about the research project.

What was good/bad about the way you were introduced to the research

Why did you agree to be a participant

Was there anything which made you consider not taking part

Experience of scan

Did you have an opportunity to discuss concerns and preferences about having the scan?

Did you have any physical and/or psychological concerns before having the scan?

Where these adequately addressed?

If so, how?

From your experience, how well was the scan appointment organised?

Did we do enough to cater for your needs e.g., physical accessibility, or around work and life commitments? i.e., were you given a choice about when the scan was done?

Did you feel that the clinical team provided a high standard of care, including quality and safety.

For example, they could answer any questions you had about the research or the FAST MRI scan

How did you feel about the amount of control you had in the lead up to and during the scan?

Did you have a chance to ask questions?

Did you feel you could stop the process?

If not, how can we make the experience better for people in the future?

Service environment and Waiting times

Can you tell me about what it was like getting to the appointment?

How did you travel? – was parking OK?

What was it like to wait for your scan? – environmental factors, psychological (nervous anxious etc), social (did you bring anyone with you; do you know anyone who has had an MRI before)

Did you have to wait long? How did that make you feel?

Acceptability

Can we now talk about how acceptable you found the FAST MRI scan.

If you were offered another FAST MRI scan would you have it?

Would this be over a mammogram?

Why?

If not, why not?

In comparison to having the mammogram how much of a burden was it to have the FAST MRI?

physical discomfort

psychological discomfort during and after the test

psychological distress experienced before it and, in the case of a negative test in particular, relief after it

Can you tell me what your understanding is of why the test is done? How should we describe the test to women in the future?

This will depend on information provision and health literacy

How easy was it for you to have the scan?

Psychological

Physical

What factors do you think we need to consider to make the test accessible for anyone to have?

Were there any economic implications for you having the FAST MRI scan?

Did you have to take time off work etc

Are there any other aspects of being part of the research or of having the FAST MRI scan that you would like to tell me about?

Personal preferences for trade-offs between over-/underdiagnosis of a condition,

attitudes to risk,

patients' preferences for body privacy have to be forgone in undergoing the test are also relevant.

Supplementary material file 4: Potential adverse effects of study intervention

- Magnetic Resonance Imaging, with and without the intravenous administration of GBCAs is carried out on patients and screening clients at hospitals across the UK every day as part of standard care.
    - FAST MRI is a component of DCE-MRI, which is a standard procedure in the UK as a breast cancer screening test for women at high risk of developing breast cancer. DCE-MRI is also used as an imaging test in standard UK clinical practice to stage breast cancer prior to neoadjuvant chemotherapy and for lobular cancers prior to surgery, to assess response to neoadjuvant chemotherapy and to troubleshoot diagnostic uncertainties.
    - Like DCE-MRI, and MRI tests routinely used to image many other parts of the body, FAST MRI involves the intravenous injection of gadolinium-based contrast agent (GBCA).
- The type of GBCA that will be used in this study is macrocyclic GBCA. The dosage will be standardised, by participant weight and height, across recruiting sites and will be the lowest dose to provide adequate enhancement of the study intervention.
- The safety profile of macrocyclic GBCA is well documented and radiology departments administer them to patients and screening clients on a daily basis in the UK.
    - GBCAs are associated with a very low rate of immediate adverse events (0.06%-0.09%),
    - Most adverse events are mild and can be managed in the radiology department.
    - Major life-threatening contrast reactions to GBCAs are extremely rare. The incidence of acute, severe reactions is estimated to be 0.0025-0.005%

- Tiny amounts of GBCA can be retained in the brain with repeated administration, although this is much less for macrocyclic than for linear GBCAs. The long-term clinical consequences of this retention are unknown although it has been extensively investigated and studies to date have been reassuring (see below).

- Guidance on GBCA administration is available from the Royal College of Radiologists website: https://www.rcr.ac.uk/publication/guidance-gadolinium-based-contrast- agent-administration-adult-patients and this guidance is followed in UK radiology departments whenever GBCAs are administered, including during research studies (*Roditi G. Guidance on gadolinium-based contrast agent administration to adult patients (Royal College of Radiologists Guidance) [Internet]. 2019. Available from:* https://www.rcr.ac.uk/system/files/publication/field_publication_files/bfcr193-gadolinium-based-contrast-agent-adult-patients.pdf)
- The European Medicines Agency has published a pharmacovigilance risk assessment report on GDCA use (*European Medicines Agency, Pharmacovigilance risk assessment committee (PRAC), Committee for Medicinal Products for Human Use (CHMP): European Commission final decision on Article 31 Referral (EMEA/H/A-31/1437). 2017 [cited 2024 May 4]. Gadolinium-containing contrast agents - European Commission Final Decision. Available from:* https://www.ema.europa.eu/en/medicines/human/referrals/gadolinium-containing-contrast-agents)
- Safety Guidance for Magnetic Resonance Equipment in Clinical Use was published by the MHRA in February 2021 and is available online: https://assets.publishing.service.gov.uk/government/uploads/system/uploads/attachment_data/file/958486/MRI_guidance_2021-4-03c.pdf
