# Supplementary material for: FAST MRI: DYAMOND trial protocol (can an abbreviated MRI scan detect breast cancers missed by mammography for screening clients with average mammographic density attending their first screening mammogram?)—a diagnostic yield study within the NHS population-risk breast screening programme
Source: BMJ Open. 2025 Sep 28;15(9):e106545. doi: 10.1136/bmjopen-2025-106545 (PMC12481369; doi:10.1136/bmjopen-2025-106545)
Supplement: Supplementary file 2 [file bmjopen-2025-106545.reviewer_comments.pdf]

PEER REVIEW HISTORY

BMJ Open publishes all reviews undertaken for accepted manuscripts. Reviewers are asked to complete a checklist review form and are provided with free text boxes to elaborate on their assessment. These free text comments are reproduced below.

ARTICLE DETAILS

Title (Provisional)

The FAST MRI: DYAMOND trial protocol (Can an abbreviated MRI scan detect breast cancers missed by mammography for screening clients with average mammographic density attending their first screening mammogram?): a diagnostic yield study within the NHS population-risk breast screening programme

Authors

Jones, Lyn Isobel; Geach, Rebecca; Loose, Abi; McKeown-Keegan, Sadie; Marshall, Andrea; Halling-Brown, Mark; Curtis, Siân; Harding, Sam; Rose, Jan; Matthews, Helen; Vinnicombe, Sarah; Shaaban, Abeer M; Taylor-Phillips, Sian; Dunn, Janet; The FAST MRI Study Group, On behalf of

---

VERSION 1 - REVIEW

**Reviewer**	**1**
**Name**	**Lau, Eddie**
**Affiliation**	**Austin Health, Department of Molecular Imaging and Therapy**
**Date**	**24-Jul-2025**
**COI**	**None**

Well done on a clearly articulated protocol paper. All the best with patient recruitment and study progress.
